# Lending a hand: supportive exercise therapy for cancer treatment-induced polyneuropathy of the upper extremity—VISCIPH A

**DOI:** 10.1007/s00520-025-09712-2

**Published:** 2025-07-22

**Authors:** Stefanie Siebert, Jane Kersten, Annika Tomanek, Sarina Heinz, Timo Niels, Freerk T. Baumann

**Affiliations:** 1 Department I of Internal Medicine, Center for Integrated Oncology Aachen Bonn Cologne Duesseldorf, University Hospital of Cologne, Kerpener Str. 62, 50937 Cologne, Germany; 2 University of Cologne, Cologne, Germany

**Keywords:** Prevention, Neuropathy-induced symptoms, Sensorimotor and vibration, Upper extremities, Feasibility

## Abstract

**Purpose:**

Chemotherapy- and immunotherapy-induced peripheral neuropathies (PNP) are common and often dose-limiting side effects of cancer treatment. Patients often experience pain, numbness, and tingling in their extremities. Pharmacological options such as duloxetine, which is recommended for chemotherapy-induced PNP (CIPN), offer limited relief [1]. Consequently, neuromuscular training incorporating sensorimotor elements is a promising non-pharmacological alternative. However, its effect on symptoms in the upper extremities remains unexplored. This study investigates the feasibility of combined sensorimotor and vibration training for the upper extremities before and during cancer treatment.

**Methods:**

The VISCIPH A study is a two-arm, prospective, randomized controlled proof-of-principle trial. Within the overarching VISCIPH A/B research framework, this is an independently designed sub-study. The primary outcome was feasibility; secondary outcomes included patient-reported outcome measures (PROMs). Participants were randomized to either a sensorimotor and vibration training group (PNPEX) or a moderate resistance exercise group (MREX). Both interventions were supervised and performed twice weekly over 12 weeks. Fine motor skills, depth sensitivity, and temperature sensation were assessed pre- and post-intervention. PROMs (EORTC-QLQ-C30, FACT/GOG-Ntx, NRS (pain)) were collected at baseline, week 4, week 8, and week 12.

**Results:**

Of 50 enrolled cancer patients, 40 completed the study (32.5% male; mean age = 50.8 years). A total of 874 out of 960 planned sessions (91%) were completed. The dropout rate was 20%, with high patient adherence (98%) and successful implementation of the extensive test battery, exhibit feasibility of the study (proof of principle). Thirteen participants reported no numbness or tingling in the hands, and 18 reported no discomfort over the 12-week period. Both groups showed significant improvements in global health status (EORTC-QLQ-C30) at T3 (*p* = 0.001). However, the MREX group showed significant deterioration in depth sensitivity at 2 of 4 bone points after 12 weeks (I CP: *p* = 0.01; III CP: *p* = 0.046), whereas PNPEX outcomes remained stable.

**Conclusion:**

While prior research has mainly focused on lower extremities, this study demonstrates the feasibility and potential protective effects of a combined vibration and sensorimotor training protocol for the upper limbs during neurotoxic cancer therapy. Given the limited efficacy of current pharmacological approaches, further research is warranted to explore the therapeutic potential of structured exercise interventions in the management of PNP.

**Supplementary Information:**

The online version contains supplementary material available at 10.1007/s00520-025-09712-2.

## Introduction

Several randomized, controlled studies showed beneficial effects of exercise interventions on oncological patients. Reported effects range from an improved everyday life functionality to reductions in treatment-associated side effects. The level of evidence varies widely [2]. As a result, in 2018, a panel of experts led by the American College of Sports Medicine (ACSM) updated the exercise guidelines for cancer survivors to reflect emerging evidence. The revised recommendations were structured according to the FITT principle: Frequency, Intensity, Time, and Type. While exercise therapy is supported by strong evidence as a safe and beneficial supportive treatment in oncology, uncertainties persist regarding its optimal use during on-going treatment, dosing, and across different cancer types. Exercise therapy has established itself as a safe and effective supportive therapy in oncology [3].

Polyneuropathy (PNP) is one of the most common treatment-related side effects in oncology, frequently caused by neurotoxic therapeutics. Although the underlying pathophysiological mechanisms vary depending on the drug class, common pathways have been identified. Depending on the agent, neuronal damage may result from axonal degeneration, mitochondrial dysfunction [4], or oxidative stress [5]. Some substances, such as platinum-based compounds, induce direct DNA damage in dorsal root ganglia neurons [4]. Microtubule-disrupting agents like taxanes impair axonal transport [6]. Immunotherapy-related neuropathies are typically immune-mediated, involving inflammatory responses triggered by T-cell activation and cytokine release [7]. These mechanisms can lead to both sensory and motor deficits, with symptoms often persisting long after treatment completion. The initial symptoms present as numbness and/or tingling sensations, known as sensory dysesthesia. In addition, motor and autonomic nerves may also be affected.

Chemo- and immunotherapy-induced polyneuropathy can be a major reason for delaying therapy or dose reductions [8]. A substantial advance would be the ability to protect patients from the onset of such burdensome symptoms and the resulting restrictions in everyday life during medical treatment [9].

To date, there are no clinically effective strategies to prevent development of PNP. However, exercise therapy has shown positive effects on PNP-related sensory and motor symptoms [10, 11]. Papadopoulou et al. (2023) summarize alternative treatments for PNP symptoms in a meta-analysis to examine their effectiveness on pain and quality of life (QoL). Acupuncture, exercise, and yoga provided PNP-related pain relief, whereas pharmacological treatments showed no effects [12, 13].

With regard to chemo- and immunotherapy-induced polyneuropathy, the German S3 guideline “Breast cancer” (2021) recommends vibration, balance (sensorimotor), and strength training [14]. Recent studies suggest that vibration training enhances sensory processing and facilitates the activation of motor units by stimulating proprioceptive afferents [15]. In addition, it may improve peripheral blood flow and support neuroplastic adaptations in the central nervous system, contributing to functional recovery in patients with sensory and motor deficits [16]. Furthermore, in their systematic review, Streckmann et al. (2014) found that, for toxically induced neuropathies, balance exercises were particularly effective in alleviating both motor and sensory symptoms. These interventions were also associated with improvements in gait stability [10]. Nonetheless, published evidence for effects of exercise therapy is not sufficient, according to ACSM guidelines [3].

It is of great interest to develop further study protocols to determine the effectiveness of different training models and to assess whether exercise can be a potential treatment option for PNP. A combination of the aforementioned training with sensorimotor and/or vibration training promotes a balanced and coherent overall training concept [17]. Most studies primarily report on the effectiveness of exercise therapy in relation to peripheral polyneuropathy of lower extremities [10, 11]. As the functional impairment can also affect upper extremities, evidence-based results are therefore required. Only two studies shed light on exercise therapy with regard to drug-induced dysfunctions of the upper extremity [18, 19]. Moreover, only one study to date has demonstrated that neuromuscular training can reduce the incidence of therapy-induced peripheral neuropathy by 50–70% [20].

The aim of the present study is to investigate whether a training intervention has a preventive effect on symptoms of PNP in upper extremities. The focus is on the feasibility of such supportive exercise therapy as proof-of-principle in the setting of impending neurotoxic therapy in oncology patients.

## Methods

### Subjects

This randomized, controlled pilot study was the preliminary research for a monocenter prospective trial, comparing a 12-week exercise intervention with an active group. The trial was registered in the German Clinical Trials Register on 10/15/2020 (DRKS-ID: DRKS00023287) and was not externally funded. The Ethics Commission of the Medical Faculty at the University of Cologne (reference number 17–165) approved the study.

The study was conducted at the University Hospital of Cologne in the Center for Integrated Oncology (CIO). Patients’ inclusion criteria were as follows:

1. Cancer diagnosis,
2. Age 18 and above,
3. Scheduled to receive first-time neurotoxic chemo- or immunotherapy,
4. Informed consent,
5. Written clearance from treating physician.

Patients were included a maximum of four weeks after the first administration of neurotoxic anti-cancer therapy. Neurotoxic potential is primarily associated with taxanes, platinum compounds, vinca alkaloids, epothilones, bortezomib, and immune checkpoint inhibitors such as nivolumab and pembrolizumab. Participants were assessed for eligibility between September 2020 and March 2023. Pre-existing polyneuropathy due to diabetes or alcohol abuse was an exclusion criterion without a neurological examination. Furthermore, the presence of a disease that precludes participation in a sports intervention or the presence of contraindications to vibration training were exclusion criteria, namely, osteolysis, osteosynthesis, bone metastases at risk of fracture, acute thrombosis, cervical disc prostheses, or total joint arthroplasty.

**Fig. 1:**
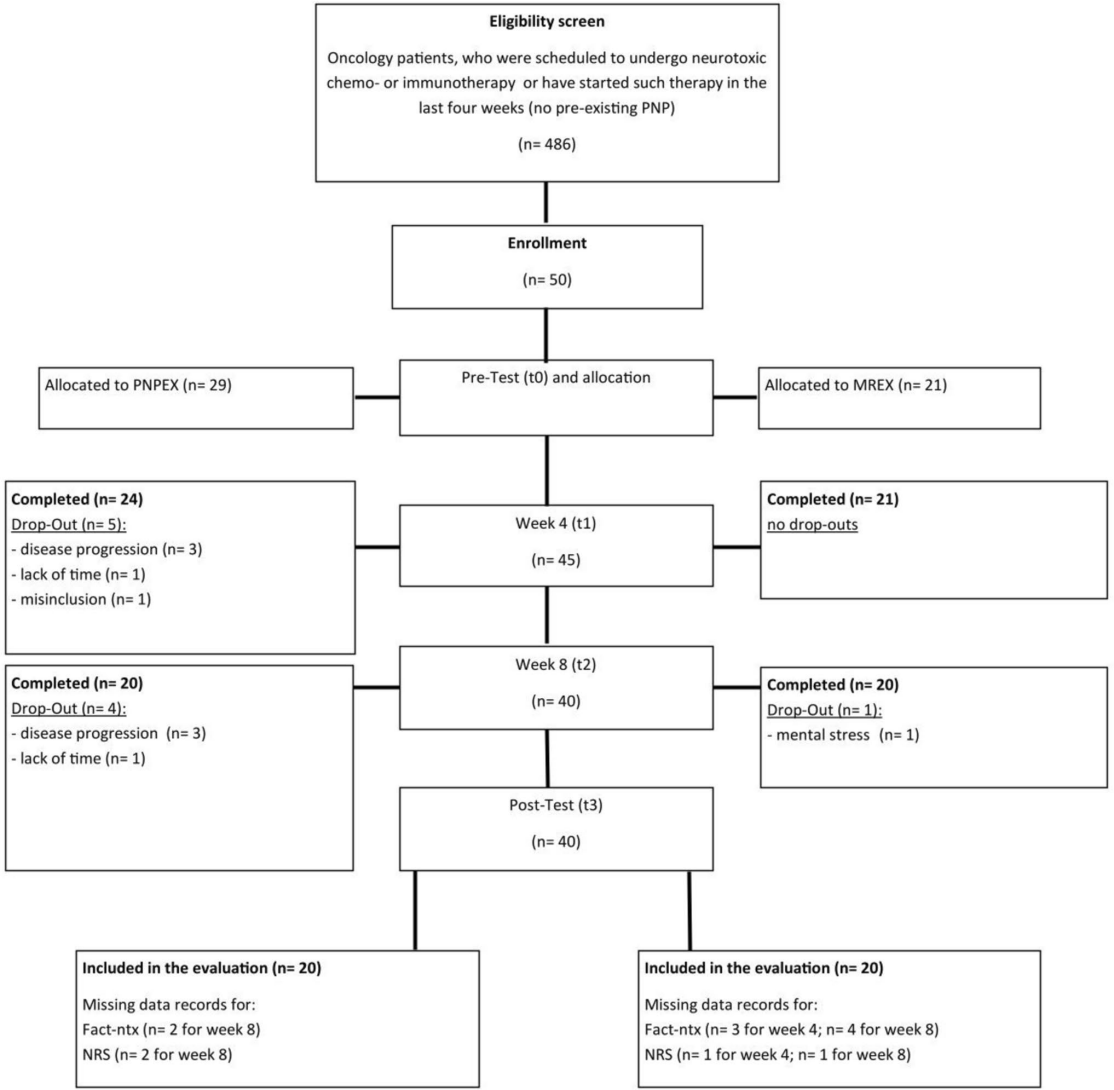
Flowchart VISCIPH A

Patients who met inclusion criteria and agreed to participate in the study were randomly assigned to one of two groups using a computer-generated randomization schedule; participants were blinded to group allocation: PNP-exercise intervention (PNPEX) and moderate resistance exercise intervention (MREX) (Fig. 1).

### Exercise intervention

Both groups underwent standardized exercise therapy twice a week for a maximum of 60 min per session. The intervention period lasted 12 weeks and was supervised by qualified therapists and carried out on the exercise area of the oncological training therapy (OTT). The intervention began with a 10-min warm-up program on cycle ergometer or elliptical trainer. The load was set to RPE 5–6 (moderate intensity), followed by 30–45 min of group-specific training. If participants have not completed at least 50% of the intervention’s total scope, this exercise session will not be deemed complete.

Patients in PNPEX received a combined sensorimotor and vibration training for upper extremities. A Galileo Fit side-alternating vibration plate (type no. 8N057410) was used for the vibration training. Based on previous study protocols, the vibration training started at 18 Hz and lasted 30 s [21]. After each training session, the frequency was increased by 1 up to 36 Hz. The recovery time should always last at least as long as the exercise time and was used to integrate the sensorimotor training. Different exercise variations were summarized in advance and categorized according to difficulty. By using resistance and a balance board, exercises could be made even more difficult and coordination could be trained simultaneously. The sensorimotor training between the sets of vibration training was carried out with everyday objects. The supplementary files contain a comprehensive description of the intervention.

The MREX group performed moderate training on six different strength training machines. Perceived exertion was assessed using the RPE scale and was intended to be between 5 and 6. Two sets of 20 repetitions were performed at 50–60% of 1RM with a 60-s rest between sets, using the following machines: total abdominal, low row, chest press, lower back, leg curl, and leg press. Finally, both groups completed a 10-min endurance session on the above machines at RPE 5–6, indicating moderate intensity.

### Measurements

Objective diagnostics were carried out before and after the 12-week training intervention in order to compare the effects of the two protocols. In addition to the pre- and post-tests, the patients completed paper-based questionnaires on-site at weeks 4 and 8 (T0–T3) to reassess subjective symptoms of PNP.

### Primary goals

The main objective of this pilot study was to determine the feasibility of using exercise to support the treatment of drug-induced polyneuropathy of the upper extremities. This included assessing serious adverse events (SAEs), dropout rates, the implementation of the test battery, and achievable training volumes.

### Secondary goals

Another objective was to reduce the incidence of PNP after completion of the intervention, as determined by objective and patient-reported outcomes. The following assessments were used to determine the incidence and severity of PNP:

1. Peripheral depth sensitivity was assessed with a Rydel-Seiffer tuning fork (128 Hz) on a graded scale from 0 (no sensitivity) to 8 (highest sensitivity) [22]. The measurement was taken on both extremities at the following measuring points: ulnar styloid process, I, III, and V carpometacarpal joint).
2. Health-related QoL was measured using the EORTC-QLQ-C30 questionnaire [23]. Cronbach’s *α* lies between 0.72 and 0.95, whereby there are no universal cut-off values.
3. Subjective report of neuropathic symptoms in the upper extremity: evaluated with a visual analog scale (VAS), 0 (not at all) to 10 (very strong). VAS is considered valid and reliable for pain assessment, with high sensitivity to change [24].
4. Patient report PNP-related symptoms using the FACT/GOG-Ntx questionnaire [25]. Cronbach’s *α* ranges from 0.82 to 0.89, and the clinically significant difference (MCID) ranges from 1.38 to 3.68 points. The questionnaire contains two specific questions about symptoms in the hands, which are examined:
5. Ntx1: “Do you experience numbness or tingling in your fingers or hands?”
6. Ntx3: “I have discomfort in my fingers or hands.”

### Statistical analysis

Statistical analyses were conducted using SPSS Statistics version 26 (IBM Corp.). Normality was assessed using the Shapiro–Wilk test and by visually inspecting *Q*–*Q* plots. Parametric or nonparametric tests were applied depending on the distribution. Descriptive statistics were calculated for all variables. Pre/post-comparisons and group effects were analyzed using mixed-design ANOVAs with repeated measures. The development of PNP, independent of group allocation, was also examined using a multifactorial repeated-measures ANOVA. A significance level of *p* < 0.05 was applied.

Missing data were evaluated and handled based on their extent and pattern. Multiple imputation was performed if the missing data were at random; otherwise, complete-case analysis was used. Figure 1 supplies details.

## Results

### Study population and characteristics

Fifty cancer patients agreed to participate in this study and underwent randomization, as well as baseline assessment. One participant was excluded from the analysis due to an incorrect inclusion due to underlying disease by the study management and did not commence the exercise intervention; another nine participants dropped out (Fig. 1).

Overall, 40 participants completed the intervention and were evaluated (Table 1). There was a maximum of 6 months between the initial cancer diagnosis and the start of the intervention. Thirty-one of 40 participants were treated with chemotherapy. A total of 24 participants (60%) received neurotoxic therapy that included either taxanes, platinum derivatives, or a combination of both (for details, see Table 1).

**Table 1:** Baseline demographics

Patients(*n*=40)									
*Gender*	PNPEX(*n*=20)					MREX(*n*=20)			
Female	15 (75%)					12 (60%)			
Male	5 (25%)					8 (40%)			
	M ± SD		Min	Max		M ± SD	Min	Max	*p*-value
*Anthropometric data*									
Age (year)	50.5 ± 13		25	80		50.7 ± 14	23	74	0.991
BMI (kg/m^2^)	24.3 ± 3.3		18	32		24.7 ± 5.3	18	39	0.762
*Disease characteristics*									
*Cancer sites*									
Breast	9 (45%)					6 (30%)			
Multiple myeloma	1 (5%)					1 (5%)			
Pancreas	1 (5%)					0 (0%)			
Hodgkin’s lymphoma	3 (15%)					2 (10%)			
Lung	0 (0%)					1 (5%)			
Prostate	0 (0%)					1 (5%)			
Gastrointestinal	2 (10%)					5 (25%)			
Gynecological	2 (10%)					1 (5%)			
Urological	1 (5%)					0 (0%)			
Cutaneous malignancies	0 (0%)					2 (10%)			
Soft tissue sarcomas	1 (5%)					1 (5%)			
*Chemotherapy*									
Only taxanes	7 (35%)					7 (35%)			
Only platinums	3 (15%)					3 (15%)			
Only vinca alkaloids	4 (20%)					3 (15%)			
Taxanes and platinums	2 (10%)					2 (10%)			
Inhibitors*	2 (10%)					2 (10%)			
Immunochemotherapy**	2 (10%)					3 (15%)			

*Carfilzomib, bortezomib, thalidomid, nivolumab, trastuzumab

**Paclitaxel + trastuzumab + pertuzumab; carboplatin + paclitaxel + bevacizumab; platinums and pembrolizumab

The inferential statistics showed no significant group differences regarding the parameters of gender distribution, age, training adherence, and BMI (Table 2).

**Table 2:** Inferential statistics of the group sample

*Dependent variable*	df	*F*	*p-value*
*Gender*	38	3.709	0.324
*Age*	38	0.176	0.991
*BMI*	38	0.855	0.762
*Group assignment*	38	2.507	0.123

### Primary goals

The primary aim was to determine the feasibility of a 12-week preventive exercise approach for the upper limb. A total of 486 patients were screened for eligibility and 130 were invited to participate. Fifty cancer patients participated in the VISCIPH A trial before starting their medical therapy. Of the 50 participants, 20% (*n* = 10) had to discontinue participation (Fig. 1). Out of 960 planned exercise sessions, 874 (91.0%) were completed (Fig. 1). The exercise compliance within the group is as follows: MREX = 93.2%; PNPEX = 89.1%. The differentiated assessment was completed 862 times, corresponding to a total of 97.4%.

A total of 22 questionnaires were found to be missing over the 12-week period due to non-participation in the exercise session.

In the investigation of the pre- and post-therapy measurements, the extensive test battery showed a participation rate of over 99%. Only one hand-grip-strength measurement could not be performed by a single patient due to the underlying disease. Neither during the intervention nor during the tests serious adverse events were reported within the VISCIPH A study.

### Secondary goals

#### Intragroup comparison: EORTC-C30, NR-scale pain, depth sensitivity

The numeric rating scale showed a mean increase in pain symptoms of 0.95 across both groups over the course of the intervention (PNPEX and MREX), with no significant difference between the two groups (*p* = 0.059; 95% CI 0.93–0.95).

Pain, physical functioning, and global health status (GHS) were also evaluated using the EORTC-QLQ-C30 questionnaire. Pain symptom burden decreased in the MREX group (mean change = − 11.67), while it increased in the PNPEX group (mean change = + 7.66); however, neither change reached statistical significance. However, both groups demonstrated a statistically significant improvement in global health at T3 (*p* = 0.001). Physical functioning declined slightly in the MREX group (by 2 points), while it remained stable in the PNPEX group (+ 0.67).

**Fig. 2:**
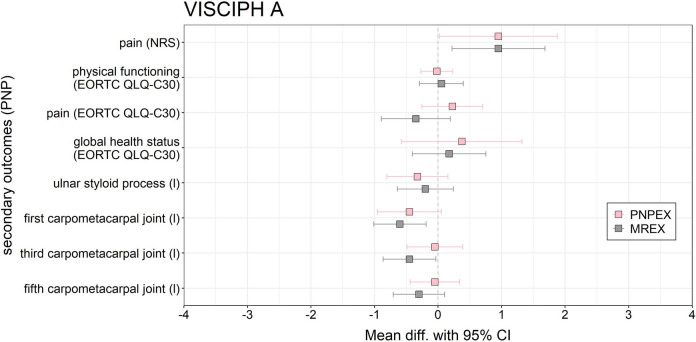
Forest plot of the effects of PNPEX vs. MREX on secondary outcomes on PNP for VISCIPH A. Data are reported as mean difference (pre-post-comparison) (95% confidence limits)

Regarding objective sensory testing, the MREX group exhibited significant deterioration in pressure sensitivity at two anatomical sites: the first and third carpometacarpal joints (mean change = − 0.6 and − 0.45 respectively, 95% CI 0.41, *p* = 0.010 and *p* = 0.046 respectively). No significant changes were observed in the PNPEX group (Fig. 2).

Of the 16 secondary endpoints assessed, only four reached statistical significance. Both groups showed clear improvements in global health, while deterioration in sensory function was limited to the MREX group. Overall, overlapping confidence intervals suggest minor trends without one intervention being clearly superior to the other (Fig. 2). A detailed summary of all outcomes, including pre-post comparisons of PROMs and performance-based measures per group, is presented in Supplementary.

#### FACT/GOG-Ntx items [1 and 3]

**Fig. 3:**
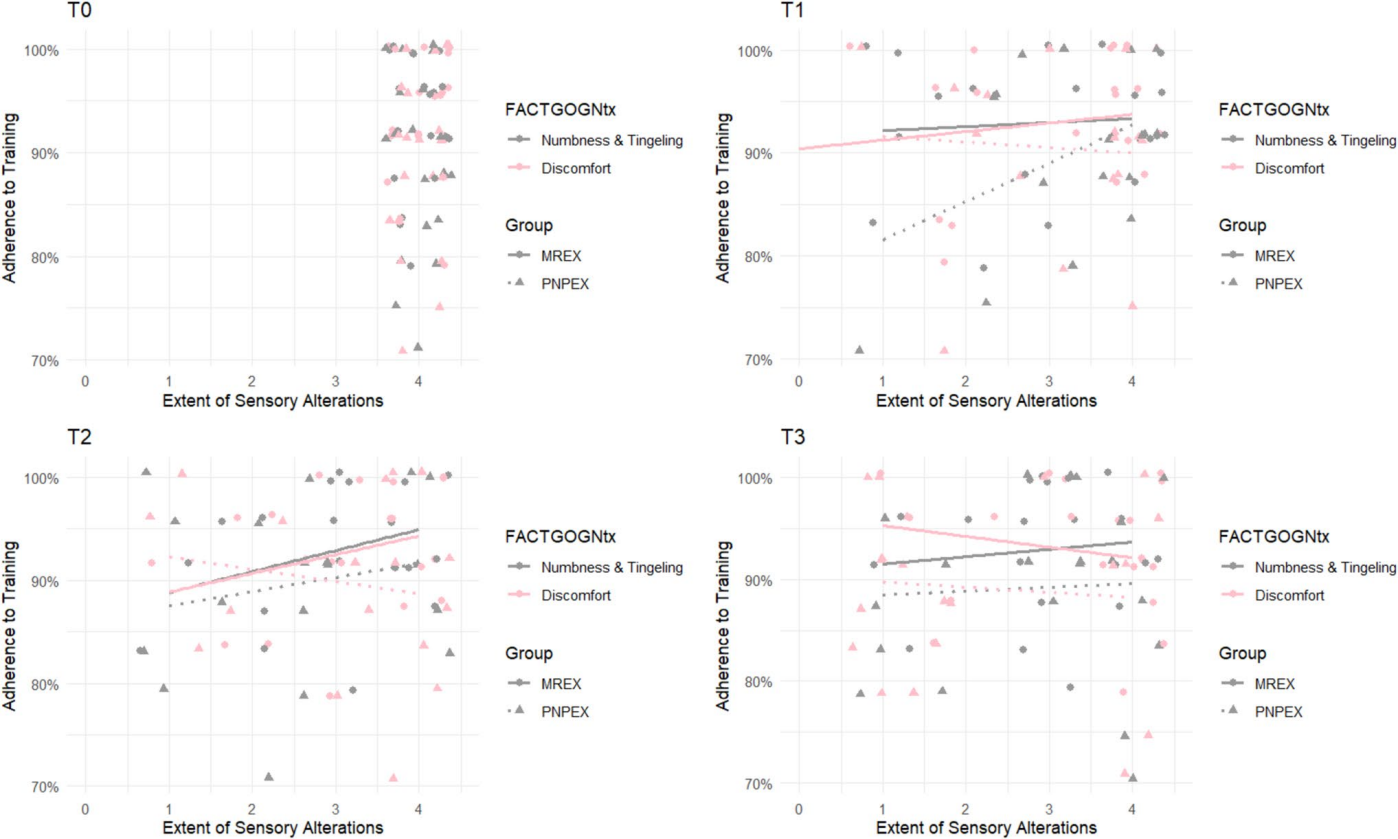
Correlation between items 1 and 3 of the FACT/GOG-Ntx and training adherence. The graph shows how numbness or tingling in the hands (item 1, grey) and discomfort in the hands (item 3, pink) developed over time for all participants (n = 40). Group affiliation is indicated by symbols: circles for MREX and triangles for PNPEX. The x-axis shows the 5-point Likert scale (0 = not at all to 4 = very much), with higher values indicating a better quality of life

At baseline (T0), all participants reported a score of 4 on item 1 (“numbness or tingling in the hands”), indicating an absence of symptoms. After 4 weeks, almost half of the participants still showed no signs of sensory polyneuropathy. The mean scores at T1 were 3.23 (PNPEX) and 3.01 (MREX). By T2, the distribution had shifted further toward lower values, with 28.8% of participants reporting scores of 1 or 2. The mean scores decreased to 2.79 (PNPEX) and 2.91 (MREX). However, by week 12, 75% of participants reported scores in the range of 3 or 4, suggesting a low increasing symptom burden (Fig. 3). A pre-post analysis revealed a statistically significant deterioration in both groups (PNPEX: *p* < 0.01; MREX: *p* < 0.01). There was no significant time-by-group interaction (*F*(3, 87) = 0.40, *p* = 0.75).

Item 3 (“discomfort in the hands”) followed a similar trend. In the MREX group, symptom scores decreased by approximately 25% at T1, remained stable, and then increased to 3.29 at T3. PNPEX participants reported a mean score of 2.93 at T1 and T2, followed by a further increase at T3. Both groups showed a statistically significant worsening over time (PNPEX: *p* < 0.01; MREX: *p* = 0.03). Again, no significant interaction between time and group was observed (*F*(1.92, 53.72) = 2.50, *p* = 0.09).

Throughout the entire intervention period, 18 participants remained free of hand discomfort and 13 showed no symptoms of numbness or tingling (Fig. 3).

## Discussion

VISCIPH-A investigated the preventive effect of PNPEX in patients starting neurotoxic cancer therapy. Literature shows that supervised exercise can preserve depth sensitivity and minimize limitations of PNP [20]. In our study, the trainability of cancer patients—understood as their capacity to engage with and respond to structured exercise programs during therapy—and the feasibility of combined sensorimotor and vibration training for upper extremities can be confirmed. It was shown that an exercise program is possible during neurotoxic therapy and that the range of symptoms remains low. The drop-out rate is similar to comparable intervention protocols involving patients with breast or gynecological cancer undergoing neurotoxic treatment [19, 26].

Moderate strength training leads to no changes in the individual’s prevalent polyneuropathy [11, 27]. When strength training with resistance bands and gait training on different surfaces is performed, one study in colorectal cancer patients shows positive effects on PNP symptoms, which are probably due to sensorimotor components of these training methods [18]. The present study design was chosen with regard to the selection of an active control group (MREX), as previous studies could not prove the effectiveness of strength training on existing PNP [11].

The effect of exercise on upper limb sensory function is represented by the prevention of deterioration of vibratory sensation. While the vibration sensation of the MREX group decreased significantly, that of PNPEX remained constant. There is evidence that an exercise-induced increase in blood flow increases the overall metabolic rate, which is associated with higher levels of neurotrophic factors that can influence sensory function. In addition, vibration training directly affects the nervous system by inhibiting spinal reflex excitability [28]. The present results support this indication and it can be assumed that targeted training similar to PNPEX may have a preventive effect. For the MREX group, literature was consulted to discover how sporting activities influence polyneuropathies. Kneis and colleagues (2019) were able to show a deterioration of - 16.9% [19] in the control group with regard to depth sensitivity. Accordingly, our data show a significant reduction in depth sensitivity at the first and third carpometacarpal joint. In comparison to previously reported inactive groups, it can be assumed that moderate strength training attenuates symptoms. Overall, exercise seems to be superior to inactivity in regard to maintaining depth sensitivity.

Looking at the results of the EORTC-QLQ-C30 while taking clinical relevance of the change (differences of ≥ 7 points) into account [29], the VISCIPH A study can show that there is a clinically relevant change despite the cancer therapy. PNPEX maintained stable physical function scores over 12 weeks and showed a significant improvement in GHS (*p* = 0.001). MREX showed a clinically relevant change in the pain level in pre-post comparison (T0: M = 40; T3: M = 28.3).

Pain intensity for both groups is rather low, whereas high mean values result from individual extreme cases. Evaluating the VISCIPH A study, 30% of PNPEX showed a minimum pain symptom value of 1. Pain symptoms remained stable in both groups over the course of time, therefore did not increase despite the neurotoxic therapy. In a comparable study by Hammond [27], 49% of the inactive group had an increased value of at least 1 on the NR scale after 8 weeks. In both intervention groups, there was a significant increase in pain symptoms from T0 to T1. Such an above average development of pain is also described by Loprinzi [30]. The pain intensity observed in the first cycle did not correlate with subsequent cycles. Courneya points out that the upregulation of neurotrophic factors is not only related to nerve regeneration, but could also be involved in neuropathic pain [31]. The pain syndrome could serve as an indicator of need for prophylactic treatment. This could prevent the development of taxane-induced neuropathy or at least minimize the associated symptoms. The MREX group performed better, with pain scores of less than 1. A correlation between group membership and pain development over time could not be confirmed. To be able to define whether vibration training has had a positive influence on the course of pain intensity [15], a longer observation period should be implemented in the future.

Over time, there is a significant increase in symptom burden both in the total score of the FACT/GOG-Ntx. Clinically significant changes in FACT/GOG-Ntx are not shown in the literature [32]. A previous study found that cancer patients (ovarian cancer) with known CIPN had a 10% lower FACT/GOG-Ntx score than patients who had not received chemotherapy [33]. Ajewole described clinically relevant change in PNP of - 10% [34]. In the total score of the questionnaire, 17 out of 40 of our patients exceeded this threshold. A total of 42.5% of the subjects exhibited PNP symptoms, 25% less than in the study by Ajewole et al. In 67% of adults with new-onset CIPN, clinically relevant CIPN was detected within 12 weeks of starting neurotoxic chemotherapy [34]. On average, the ratio of 91% of training sessions completed is impressive, considering that all patients were undergoing chemotherapy or immunotherapy during the intervention. The group of Bland, among others, shows a participant’s attendance of 78 ± 23% during taxane-induced chemotherapy [35]. The compliance of our intervention groups can be rated as high and shows the feasibility of the intervention. Available data suggests that the supportive therapy enables almost 1/3 of patients to complete the treatment without sensory deficits and as many as 45% to develop no pain symptoms at all. Regardless of group affiliation, both training methods have a positive influence on subjective and objective symptoms of polyneuropathy. Oncological patients can benefit from the supportive use of physical activity with regard to their polyneuropathy.

The concept of the pilot study confirms the idea of prevention after the initial onset of pain, although it can be assumed that drawing attention to PNP may actually trigger symptom awareness [36]. For this reason, the question arises as to whether it is advantageous to take preventive action against PNP pain or whether targeted training should only be started when cancer patients express symptoms. In either case, it might be worth considering general strength and endurance training in order to maintain QoL and general physical performance. The assumption that both training methods have a preventive influence on symptoms in cancer patients undergoing neurotoxic chemotherapy or immunotherapy seems likely. A recent meta-analysis supports the use of combined exercise interventions to improve quality of life and physical function in patients with CIPN, despite limited effects on sensory symptoms [37]. Potential mechanisms leading to the benefit of exercise intervention for PNP symptoms include possible anti-inflammatory effects and effects on the central nervous system [38, 39]. Streckmann showed that PNP did not improve with sensorimotor training alone, whereas a combination of muscle strengthening, balance exercises, and nerve gliding exercises achieved significant effects [10]. These results suggest that the type of exercise intervention may play a critical role, as the neuromuscular system adapts specifically and progressively to the initial training volume and intensity applied [40]. The results of the present study support this assumption. As shown in the literature, both exercise modalities show positive outcomes compared to inactive group, and both are effective in controlling the symptoms of PNP. The results suggest that exercise interventions are beneficial for cancer patients by reducing upper extremity PNP. According to the consensus-based recommendation of the German S3 guideline “Supportive therapy for oncology patients,” movement therapy should be used to improve functionality in manifest PNP [41]. In terms of future standards for supportive measures, a combination of these exercise protocols (including muscle strengthening, balance exercises, and vibration training) should be considered.

### Limitations

In order to advance future research to reduce drug-induced polyneuropathy, the limitations of this pilot study must be listed. Although significant differences could be generated in some cases despite the small sample size, a larger sample size and an inactive patient collective would be helpful.

The fact that there was no separate subdivision according to cancer type should not be ignored. Consequently, the participants were in different treatment courses and received different medication. Polyneuropathies may have developed differently depending on the neurotoxic agent and thus had an influence on the PNP or pain symptoms. In order to be able to provide a detailed statement on the effectiveness of the two training models, a subdivision of the medical therapy, or rather a differentiation according to the active substances that the therapy contains, should be carried out in the future.

## Conclusion

Exercise therapy, as a simple and feasible non-pharmacological intervention, has shown positive effects in the prevention and treatment of PNP. This study shows tendencies that both combined vibration and sensorimotor training and moderate strength training can maintain the sensory function of the hands and improve PROMs regarding the PNP-related QoL.

The exercise intervention was feasible, no adverse events were observed, and excellent compliance was achieved. This pilot study may stimulate further investigations as it provides new insights into physical exercise during neurotoxic therapy in cancer patients: The present intervention shows an improved pain level and prevention of loss of depth-sensitivity, as almost one-third of patients completed the treatment without sensory deficits. Recently, there has also been increasing evidence that more attention should be paid to non-pharmacological supportive approaches in cancer patients. At present, there is insufficient evidence to make clinical recommendations, which should be a goal of future studies.

## Supplementary Information

Below is the link to the electronic supplementary material.

Supplementary file1 (PDF 242 KB)

Supplementary file2 (PDF 221 KB)

## Data Availability

Data are available upon reasonable request.
