# Supplementary material for: Lending a hand: supportive exercise therapy for cancer treatment-induced polyneuropathy of the upper extremity—VISCIPH A
Source: Support Care Cancer. 2025 Jul 22;33(8):712. doi: 10.1007/s00520-025-09712-2 (PMC12283904; doi:10.1007/s00520-025-09712-2)
Supplement: Supplementary file 1 — Supplementary file1 (PDF 242 KB) [file 520_2025_9712_MOESM1_ESM.pdf]

PNPEX – Intervention Protocol

The intervention combines local vibration training with moderate aerobic and sensorimotor exercises. Each training session follows a standardized structure:

- Warm-up (10 min): ergometer or elliptical (corresponding to Borg scale 12-15)
- Vibration training (8 min): hands placed on a vibration plate in a kneeling position
- Sensorimotor training (15 min): functional balance and coordination exercises
- Cool-down (10 min): low-intensity ergometer or elliptical

Instructions: vibration plate

- Starting position: kneeling on a soft pad; hands placed flat on the vibration platform
- Hand placement: middle finger pointing at markings "1" through "4" in sequential order
- Weight Distribution: body shifted backward; arms relaxed, no active support through the hands

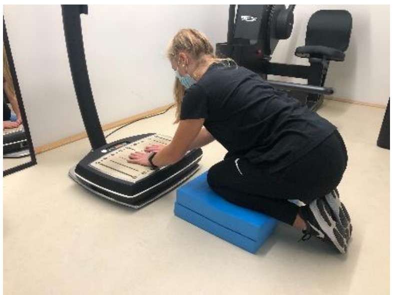

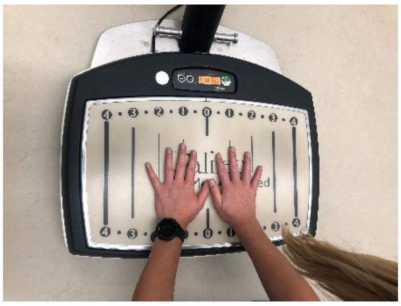

Protocol: vibration plate

Session	Frequency (Hz)	Hand Positions	Duration per Position	Pause between Positions
1	18	1–4	30 sec	60 sec
2	19	1–4	30 sec	60 sec
3	20*	1–4	30 sec	60 sec

*Progression: increase in frequency by 1 Hz per session up to a maximum of 36 Hz.

Subjective exertion was be monitored using the Borg scale (target 12–15). Adaptations were made in cases of overexertion, pain, or motor control issues.

Protocol: sensorimotor training

- Exercises per session: 3–4
- Repetitions: 3 sets
- Duration per set: 20 seconds
- Rest intervals: 20–40 s between sets; 1–2 min between exercises

Category	Exercise / Description	Material / Device	Variation / Progression
**Fine Motor Skills**	Rolling a towel, finger walking (thumb, index, middle fingers, etc.)	Towel	—
	Pinching clothespins with thumb–index / thumb–middle finger	Clothespins	10 repetitions
	Picking up cards one by one / pushing them from the hand	Playing Cards	—
	Pinching, rolling, shifting, passing cork between fingers	Cork	Individually or in series
	Collecting and stacking coins, rotating around vertical/horizontal axis	Coins	By size / axis / finger pairing
	Lacing and tying a shoe	Shoe + shoelace	—
**Grip & Coordination**	Lat pull down, biceps curl, butterfly, triceps pulldown, chest press	Resistance bands / loops	Single-arm / standing variations
	Finger trainer, squeeze ball, grip ring, resistance chain, finger extensor band	Various hand trainers	Static holds or repeated actions
**Coordination / Throwing**	Juggling, ring toss, target throwing, hand–eye coordination	Beanbags, rings, tennis balls	With movement or dual-task activities

**Balance / Stability**	Tandem / semi-tandem / single-leg stance	Mat, Airex, balance beam	With eyes closed / additional motor or cognitive tasks
	Weight shifting (forward–backward / lateral / circular)	Airex pad, Aero-Step	Add ball or visual-motor task
	Star-shaped toe taps, guiding ball with foot	Spiky ball (sensory ball)	In circles / toward a target
	Step-up (forward or lateral), maintain stance	Airex + Stepper	Single-leg, eyes closed
**Quadruped Stability**	Lifting arms / legs / diagonal combination in four-point position	Airex pad	Add theraband / slower tempo
**Thera-/Pezziball**	Sitting with eyes closed, lateral raises, trunk rotation, butterfly, rowing	Pezziball + theraband	Combine with weights
**Unstable Surfaces**	Balance board or beam tasks with ball/bag or resistance band		
