# Supplementary material for: Lending a hand: supportive exercise therapy for cancer treatment-induced polyneuropathy of the upper extremity—VISCIPH A
Source: Support Care Cancer. 2025 Jul 22;33(8):712. doi: 10.1007/s00520-025-09712-2 (PMC12283904; doi:10.1007/s00520-025-09712-2)
Supplement: Supplementary file 2 — Supplementary file2 (PDF 221 KB) [file 520_2025_9712_MOESM2_ESM.pdf]

Supplements

	PNPEX- Group					MREX – Group				
	Baseline		Post-Testung			Baseline		Post-Testung		
	Mean	SD	Mean	SD	*p*-Value	Mean	SD	Mean	SD	*p*-Value
PERFORMANCE										
20-Cent-Test										
Right hand	24.14	7.91	23.13	5.07	0.643	22.85	6.77	22.78	5.23	0.948
Left hand	24.72	5.57	24.83	6.67	0.950	23.14	5.02	24.41	5.95	0.319
Depth sensitivity										
**ulnar styloid process**										
right	6.80	0.70	6.80	0.83	1.000	6.80	0.77	6.80	0.83	1.000
left	6.90	0.91	6.45	0.89	0.095	6.90	0.72	6.70	0.80	0.385
**I carpometacarpal joint**										
right	7.10	0.91	7.05	0.76	0.825	7.20	0.77	7.10	0.85	0.694
left	7.25	0.72	6.80	1.06	0.095	7.30	0.57	6.70	0.87	**0.010***
**III carpometacarpal joint**										
right	7.15	0.67	6.95	0.76	0.385	6.95	0.76	6.90	0.91	0.834
left	7.15	0.67	7.10	0.79	0.825	7.25	0.72	6.80	0.77	**0.046***
**V carpometacarpal joint**										
right	7.20	0.62	6.85	0.75	0.090	7.05	0.61	7.00	0.65	0.772
left	7.25	0.64	7.20	0.83	0.804	7.25	0.64	7.00	0.65	0.163
Handgrip strength										
Right hand	37.75	9.05	36.46	8.10	0.105	33.47	10.12	34.18	6.82	0.725
Left hand	36.07	9.91	34.69	9.66	0.172	32.77	7.53	31.59	6.51	0.417
PROs										
Fact-GOG-ntx Score	39.75	3.82	34.05	7.61	**0.005***	38.89	4.27	36.62	5.16	0.094
BPI										
Pain Impairment	13.05	14.58	10.50	16.58	0.471	14.45	17.20	9.15	9.78	0.146
Pain Intensity	5.41	5.01	6.53	8.08	0.476	7.68	9.83	5.05	4.84	0.260
NR-Scale										
Pain	0.00	0.00	0.95	2.11	0.059	0.00	0.00	0.95	1.67	0.059
Numbness and Tingling	0.00	0.00	1.95	2.19	**0.001****	0.00	0.00	1.90	1.80	**0.001****
EORTC- C30 QoL										
Physical Functioning	80.0	19.7	80.7	17.9	1.000	79.08	22.5	77.3	19.5	0.983
Pain	24.2	21.3	31.7	39.3	0.480	40.0	40.6	28.3	28.6	0.174
Global Health State	50.0	26.2	56.3	21.9	**0.001****	51.7	17.4	54.6	19.6	**0.001****
